# Preclinical development of MK‐2214 a novel antibody targeting pS413 tau for the treatment of Alzheimer’s Disease

**DOI:** 10.1002/alz70859_102913

**Published:** 2025-12-25

**Authors:** Jonathan A Sugam, Seth Robey, Sokreine Suon, Yuan Tian, Oitak Wong, Xuemei Zhao, Ying Chen, Michael Judo, Brad E Smith, Andrei Popescu, Jonathan Kurz, W. Joseph Herring, Jeanne Baker, Sophie Parmentier‐Batteur, Rebecca M Klein, Sean M Smith, Jason M. Uslaner

**Affiliations:** ^1^ Merck and Co., Inc., Rahway, NJ USA; ^2^ Merck Sharp & Dohme LLC, West Point, PA USA

## Abstract

**Background:**

The accumulation of misfolded and hyperphosphorylated tau in the brain is a defining feature of Alzheimer’s Disease (AD) that leads to neurodegeneration and functional decline. Growing evidence supports the hypothesis that the spatial and temporal propagation of this pathology occurs via neuron‐to‐neuron spread of extracellular pathological tau species and the templating of new pathology in previously healthy cells. Antibodies targeting extracellular tau have been proposed as a therapeutic strategy to reduce the spread of pathology and slow the progression of disease. Here, we describe the preclinical characterization of MK‐2214, a novel, highly potent, half‐life extended antibody targeting phosphorylated serine 413 (pS413) containing tau species for the treatment of AD.

**Method:**

Binding assays were used to evaluate the specificity, selectivity, and potency of MK‐2214 for pS413 tau. Next, immunoassays were used to determine the potency and binding patterns of MK‐2214 for tau species present in AD patient brain and CSF. In vitro efficacy was determined using a cell‐based seeding model in which MK‐2214 immunodepleted pathological tau seeds resulted in reduced pathology in iPSC derived human neurons. In vivo studies were then conducted in nonhuman primate to determine the PK profile of MK‐2214. Finally, in vivo mouse studies were completed to evaluate the efficacy of antibody treatment on the development of pathology in a model of intracranial tau seeding.

**Result:**

These studies demonstrated that MK‐2214 is a highly potent antibody that specifically binds to pS413, a form of tau present in AD brains. Ex vivo binding assays in human CSF samples determined that MK‐2214 is effective at binding extracellular tau species at a subnanomolar potency. Further, in vitro and in vivo seeding studies showed robust reductions in tau pathology, suggesting that antibody therapy targeting pS413 tau can reduce the extracellular spread of tau seeds. Finally, PK studies in NHP demonstrated that MK‐2214 has an extended half‐life compared to traditional antibodies. Taken together these data support a favorable therapeutic profile for MK‐2214.

**Conclusion:**

The preclinical profile presented here supports the clinical development of MK‐2214 as an optimal tau antibody to slow the progression of AD.

